# Medicinal Plants Used for Eye Conditions in Mexico—A Review

**DOI:** 10.3390/ph16101432

**Published:** 2023-10-09

**Authors:** Anuar Salazar-Gómez, Amabile A. Velo-Silvestre, Angel Josabad Alonso-Castro, Luis Fernando Hernández-Zimbrón

**Affiliations:** 1 Laboratorio de Investigación Interdisciplinaria, Área de Optomtería, Escuela Nacional de Estudios Superiores Unidad León, Universidad Nacional Autónoma de México (ENES-León UNAM), Blvd. UNAM 2011, Guanajuato 37684, Mexico; asalazarg@enes.unam.mx; 2 Clínica de Optometría, Escuela Nacional de Estudios Superiores Unidad León, Universidad Nacional Autónoma de México (ENES-León UNAM), Blvd. UNAM 2011, Guanajuato 37684, Mexico; avelos@enes.unam.mx; 3 Departamento de Farmacia, Universidad de Guanajuato, Noria Alta, Colonia Noria Alta Guanajuato, Guanajuato 36250, Mexico

**Keywords:** eye conditions, ethnobotanical, medicinal plant, Crassulaceae

## Abstract

Medicinal plants have been historically significant for treating common human diseases in Mexico. Although some ethnobotanical research exists, limited ethnomedicinal data has documented medicinal plants employed for eye health. This review focuses on ethnomedicinal information and preclinical and clinical studies regarding medicinal plants used in Mexico for treating symptoms associated with eye conditions. An electronic database search was conducted by consulting scientific articles, books about Mexican herbal medicine, and academic theses. This work recorded 69 plant species belonging to 26 plant families, especially plants from the Crassulaceae family, which are used as remedies for irritation and infections in the eye. Eight of these medicinal plants have been the subject of preclinical studies using ocular models, and one medicinal plant has been tested in clinical trials. The evidence of pharmacological effects indicates the promising therapeutic potential of these medicinal plants for developing new treatments for eye conditions. However, toxicological studies are necessary to ensure safe application to the eye, particularly as traditional medicine continues to be relied upon worldwide. In addition, this review highlights the need to perform ethnobotanical and phytochemical studies in Mexico regarding the medicinal flora used as remedies for eye conditions.

## 1. Introduction

Eye health plays a critical role in the overall quality of life in the general population. The components of the visual system, along with its multiple functions, are frequently impacted by various health conditions, leading to the development of eye diseases. These conditions range from minor issues that typically do not cause vision impairment (e.g., dry eye, conjunctivitis, pterygium, blepharitis) to more severe ailments that can result in vision impairment or blindness, such as cataracts, glaucoma, corneal opacity, and refractive error [1]. In 2020, cataracts and glaucoma affected over 15 and 3 million adults aged older than 50 years, respectively, and were reported as the leading causes of blindness worldwide [2]. Although global approximations on the prevalence of eye conditions are scarce, the World Health Organization has estimated that the burden is disproportionately higher in low- and middle-income countries and among medically underserved populations [1]. In these countries, the initial approach to maintaining good eye health often involves self-treatment of eye conditions using traditional medicine [3,4,5]. The utilization of medicinal plants for preparing and administering ophthalmic remedies constitutes the foundation of traditional medicine in diverse cultures worldwide [6,7,8].

Herbal medicine plays a vital role in Mexico’s healthcare system. Throughout Mexican history, medicinal plants have been highly valued for their therapeutic properties in treating eye diseases [9]. The pre-Hispanic codices offer valuable insights into the botanical knowledge of the Nahuas, revealing the use of specific medicinal plants for treating eye conditions. For instance, pterygium was empirically treated with an eye drop made from the juice of *Pinaropappus roseus* (Less.) Less. is known as “chichicaquilitl” in Nahuatl, and “nubes de la córnea” (presumably corneal opacity) was treated with the juice of “tlatlayotli” [9]. The juice of the stem of a reed called “tule” or “ixtollin” was used to cure certain eye ailments. During the colonial period in Mexico, some of the earliest healthcare practices for eye conditions, including the use of medicinal herbs, were documented [10]. Alonso López de Hinojosos, a distinguished surgeon of New Spain in the second half of the 16th century, contributed significantly to the documentation of herbal medicine to treat several diseases. His work, “Summa y recopilación de cirugía con un arte para sangrar muy útil y provechosa” (Summa and compilation of surgery with art to bleed very useful and profitable), published in 1578, mentions more than 50 medicinal plants used as remedies for various diseases, including “tacamahaca” or “tequemahaca”, which is known to cure chronic eye diseases [11]. In later years, systematic documentation of botanical knowledge in Mexico gained momentum with the ethnobotanical movement in the 1960s and early 1970s, leading to field studies that provided valuable insights [12].

Traditional medicine continues to be prevalent in many rural areas, and researchers possess well-documented ethnobotanical data on medicinal plants used to treat common human diseases in various regions of Mexico [13,14]. However, efforts to document specific traditional knowledge of eye conditions are scarce. Only two studies on ethnobotanical information regarding eye care have been conducted worldwide, in Navarra, Spain [7] and Noakhali District, Bangladesh [15]. Therefore, this review aims to identify and organize the ethnomedicinal information available in the literature regarding medicinal plants used in Mexico for managing and treating eye conditions.

## 2. Results

### 2.1. Ethnobotanical Information of Medicinal Plants Used for Eye Conditions in Mexico

Since 1984, thirty-five ethnobotanical studies have been published and have mentioned at least one plant species for treating eye conditions. Most of the ethnobotanical studies were conducted in the states of Puebla and Oaxaca (Southern Mexico).

A total of 69 medicinal plants, belonging to 26 plant families, were identified as remedies for eye conditions or symptoms associated with eye problems (Table 1). Crassulaceae was the most represented family, with nine species (~12.8%), followed by Euphorbiaceae (seven species; 10%), Asteraceae (six species; 10%), and Lamiaceae (six species; ~8%). The most cited plants were *Matricaria chamomilla* L. (n = 10), *Rosa × centifolia* L. (n = 4), *Euphorbia prostrata* Aiton (n = 3), and *Jatropha dioica* Sessé (n = 3). 

Medicinal plants used for treating eye conditions in Mexico involve various plant parts and preparation methods, depending on the specific eye disease or symptoms associated with eye problems. The leaves, flowers, and sap are the most used plant parts, usually applied topically as eye washes or eye drops prepared by infusion (15%), sap (8%), or by squeezing the leaves (7%) (Table 1).

The sap obtained from succulent plants, belonging to the Crassulaceae and Euphorbiaceae families, is known to store water as an adaptation to arid conditions [56,57,58]. Species belonging to the Euphorbiaceae family also produce latex used for treating eye diseases due to its antimicrobial, anti-inflammatory, and wound-healing effects [59,60]. Topical eye drops are favored for treating various ocular conditions due to their accessibility and non-invasive nature [61]. People often associate eye drops with natural plant fluids, such as squeezed sap or exudates, making them a common choice for managing anterior eye segment issues.

The medicinal plants cited in this review are used for treating eight specific eye conditions and seven symptoms associated with eye problems. However, many ethnobotanical reports lack detailed pathological information, only referring broadly to “eye problems”, “ophthalmological problems”, or “eye diseases” (Table 1). 

Many medicinal plants are used for treating eye irritation and eye infections, which can be explained since the general population quickly perceives the symptoms of these diseases, such as red, burning, stinging, and watering eyes. In certain regions of Mexico, like San Miguel Tulancingo in Oaxaca (Southern Mexico), medicinal plants are applied for treating various eye conditions similar to those cited above [31].

Eye conditions have been documented using common Spanish names or traditional medical terms, such as “Vista venteada” (blurred sight), “Carnosidad”, and “nubes”. “Carnosidad” refers to the growth of conjunctival tissue over the cornea, whereas “nubes” describes corneal opacity. Both conditions are related to ocular surface disorders, which could include pterygium or corneal leucoma, respectively. Interestingly, the term “nubes” is also used to refer to cataracts, an intraocular disease that causes blurred vision. It is important to note that there are distinctions among these terms, and they have historical roots in ancient Mexican medical literature [62]. These oral traditions and expressions are still used, which reflects their enduring significance in local communities.

**Figure 1:**
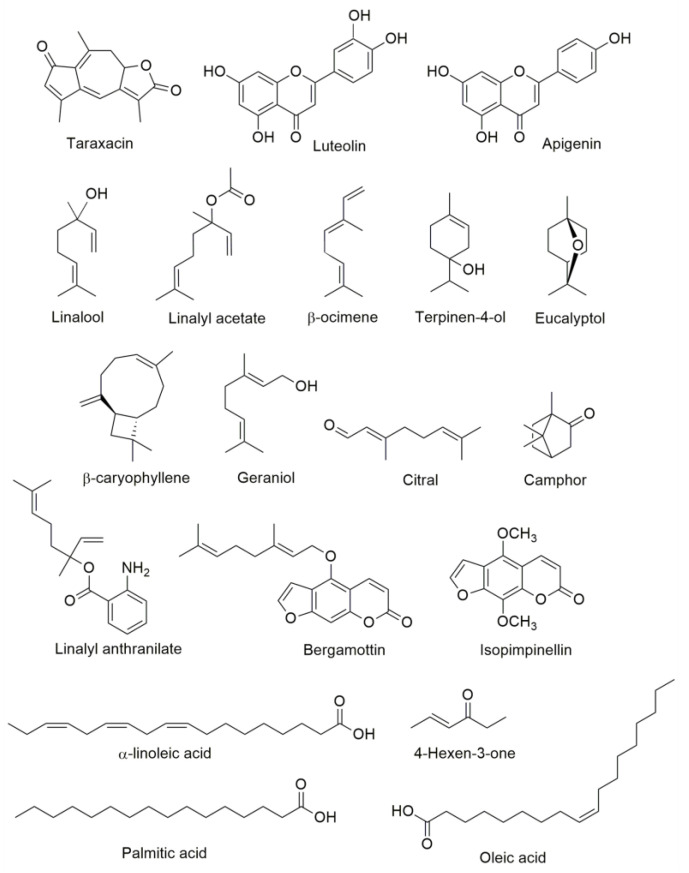
Chemical structures of compounds with antibacterial activity.

Medicinal plants used for treating conjunctivitis are administered by topical application, although some are also ingested for their potential systemic effects [63]. Conjunctivitis, a condition caused by infectious, allergic, toxic, or mechanical agents, is characterized by bulbar and tarsal conjunctiva inflammation in one or both eyes [64]. In this review, some eye ailments or symptoms related to eye problems might be associated with conjunctivitis, and their treatment involves the topical application of medicinal plants. Nerveless, *T. officinale*, *M. chamomilla*, *P. polypodioides*, *L. angustifolia*, and *C. aurantiifolia* are orally administered to treat conjunctivitis, whereas *C. nobile* and *P. laevigata* are used for eye infections. It is important to note that conventional therapy for conjunctivitis also involves the use of oral antivirals, antibiotics, and/or antihistaminic drugs, which are effective in many cases [2]. Therefore, the medicinal plants orally ingested by people could represent a promising source of new drugs to improve ocular therapy for infectious diseases—for example, the antibacterial effects of the root and aerial parts of *T. officinale* against several bacterial strains like *Staphylococcus aureus* and *Pseudomonas aeruginosa* [65]. In silico studies have revealed that luteolin and taraxacin, two components of *T. officinale,* are potential inhibitors against the methicillin-resistant gene *mecI*, a virulent factor in methicillin-resistant *S. aureus* [66]. Another example is *M. chamomilla*, one of the most common plants used for medicinal purposes. Solvent extracts of this plant and essential oils have demonstrated antimicrobial activity against several pathogens, including, *S. aureus*, *P. aeruginosa*, and *Staphylococcus epidermidis*. The antibacterial activity of *M. chamomilla* has been attributed to the presence of apigenin and α-linolenic acid [67]. *L. angustifolia* is a popular herb used to treat multiple diseases worldwide; in Mexico, oral infusions are used to treat conjunctivitis. Although an antimicrobial effect of the aqueous extract of *L. angustifolia* was not detected after the simulated gastrointestinal digestion in vitro [68], the essential oil of this plant has compounds such as linalool, linalyl acetate, β-ocimene, terpinen-4-ol, eucalyptol (1,8-cineole), camphor, β-caryophyllene, geraniol, and linalyl anthranilate that destroy the cellular membranes of pathogens and damage intracellular molecules [69]. Another example is *C. aurantiifolia*; Sandoval-Montemayor et al. [70] reported that isopimpinellin, bergamottin, palmitic acid, linoleic acid, oleic acid, 4-hexen-3-one, and citral from the hexane extract of fruit peel of this plant exert antibacterial activity against *Mycobacterium tuberculosis (*Figure 1). The bioactive compounds present in these plants may offer potential benefits for the treatment of eye conditions, and further research is needed to investigate their efficacy and safety.

Among the eye conditions reported in ethnobotanical surveys, the topical application of *P. sagittifolium* leaves to children’s eyelids during sleep for treating strabismus is particularly noteworthy, especially considering that conventional pharmacological treatment often involves the intramuscular injection of botulinum toxin as an alternative to surgical intervention [71]. Similarly, *R. communis* is used to treat strabismus in China and India [72,73]. Ricin, a toxin isolated from *R. communis* seeds, possesses ribosome-inactivating properties, making it a potential candidate for an in vivo locoregional remedy of strabismus and oculofacial dystonias. Furthermore, in vitro studies have suggested that ricin might serve as a substitute for the intramuscular injection of botulinum toxin in ocular dystonia therapy [74]. Because of its potential visual health benefits, *R. communis* has become the subject of several clinical trials. Therefore, it could be interesting to perform preclinical studies to corroborate the effectiveness of *P. sagittifolium*.

**Figure 2:**
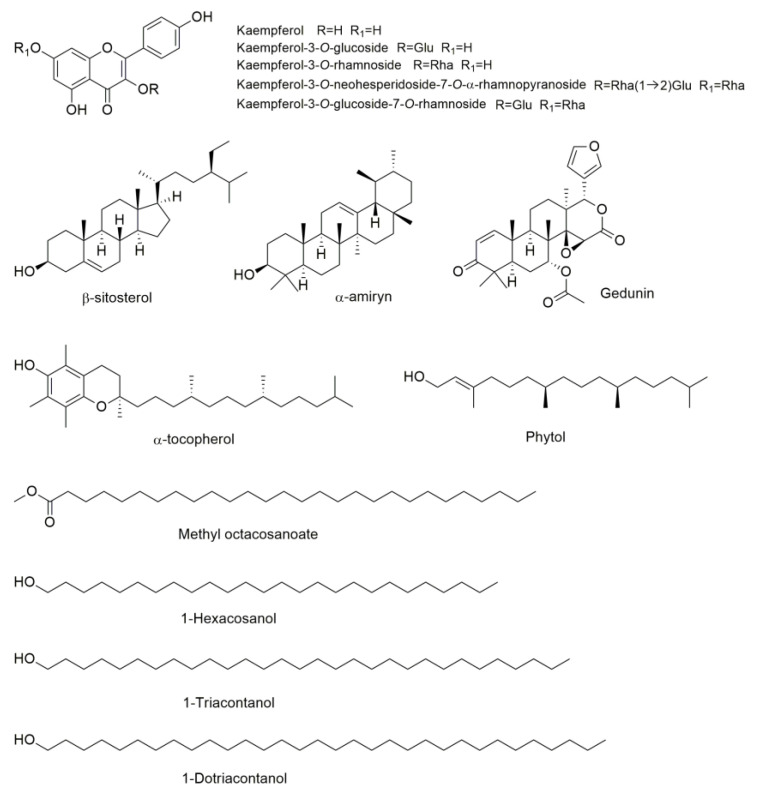
Chemical structures of some compounds found in *S. dendroideum*.

Of the 69 identified plants, 7 with ethnomedicinal information for eye conditions treatment are endemic to Mexico. Most of the species belong to the *Sedum* genus: *S. allantoides*, *S. dendroideum*, *S. diffusum*, *S. morganianum,* and *S. oxypetalum*. The *Sedum* genus, one of the most widely distributed genera within the Crassulaceae family, includes approximately 428 species [75,76], and 119 of these species are native to Mexico [77]. Many taxa of the *Sedum* genus are attractive to plant collectors due to their ornamental value, and are applied on extensive green roofs [78]. Members of the *Sedum* genus have also been reported for the empirical treatment of several diseases, such as skin inflammation, stomach pain, ulcers, hemorrhoids, and constipation [79,80,81,82]. Some of the *Sedum* species listed in Table 1 have been frequently used in Mexico for the treatment of gum diseases (*S. dendroideum*) [83], burns, skin infections, and mouth infections (*S. oxypetalum*) [84]. The dichloromethane extract of *S. dendroideum* promoted corneal healing in a murine model of pterygium-like eye lesions and exhibited immunomodulatory effects by reducing the levels of pro-inflammatory cytokines TNF-α and IL-1α, maintaining the expression of the anti-inflammatory cytokine IL-10. Moreover, the lyophilized sap of *S. dendroideum* showed antiproliferative activity in human pterygium fibroblasts and decreased the expression of vascular endothelial growth factor (VEGF) and connective tissue growth factor (CTGF), two of the most important proteins implicated in angiogenesis [85,86]. The main components of the lyophilized sap of *S. dendroideum* are kaempferol glycosides, such as kaempferol-3-*O*-glucoside, kaempferol-3-*O*-rhamnoside, kaempferol-3-*O*-neohesperidoside-7-*O*-α-rhamnopyranoside, and kaempferol-3-*O*-glucoside-7-*O*-rhamnoside (Figure 2). It has been demonstrated that kaempferol inhibits angiogenesis by decreasing VEGF expression in human ovarian cancer cells [87], and decreases the expression of VEGF mRNA in H_2_O_2_-treated ARPE-19 cells and the VEGF in sodium iodate-induced retinal degeneration in rats [88]. The main components found in the dichloromethane extract of *S. dendroideum* are 1-triacontanol, β-sitosterol, α-tocopherol, α-amiryn, methyl octacosanoate, phytol, gedunin, hexacosanol, and 1-dotriacontanol [85] (Figure 2). Torrescano-De Labra et al. [85] suggested that gedunin is associated with the inhibition of the expression of proangiogenic growth factors and cytokines and the induction of angiogenesis inhibitors such as IL-10, whereas triacontanol and α-amyrin are involved in the autoinflammatory effect. These studies suggest that *S. dendroideum* holds potential as a therapeutic agent for managing pterygium and other ocular conditions.

It is worth noting that Mexico stands out due to having a significant number of *Sedum* species that have been traditionally used for treating eye conditions. The prominence of *Sedum* species is not only due to their frequency of being reported for eye treatments, but also because they are readily available as ornamental plants. Their widespread availability makes them a feasible and accessible resource for traditional medicine practices.

### 2.2. Pharmacological Efficacy and Safety Studies

Eight of the seventy plant species used for eye conditions in traditional Mexican medicine have been examined in preclinical studies using ocular models, and one plant species has been tested in clinical trials (Table 2). Most plant extracts are prepared using water or methanol, and only *A. vera* follows the information reported by Mexican ethnomedical claims for treating eye conditions. Species belonging to the Crassulaceae and Euphorbiaceae families lack pharmacological studies using crude sap or exudate directly squeezed from the leaves.

Most of the cited preclinical models were naphthalene- and selenite-induced cataracts, followed by water-loading and steroid-induced elevated intraocular pressure (IOP) and alkali-burned corneas. This can be explained, since cataracts and glaucoma are two leading causes of blindness [2]. Natural products have demonstrated promising therapeutic effects against cataracts and glaucoma [104,105]. Therefore, efforts to investigate new therapeutic strategies are needed.

**Figure 3:**
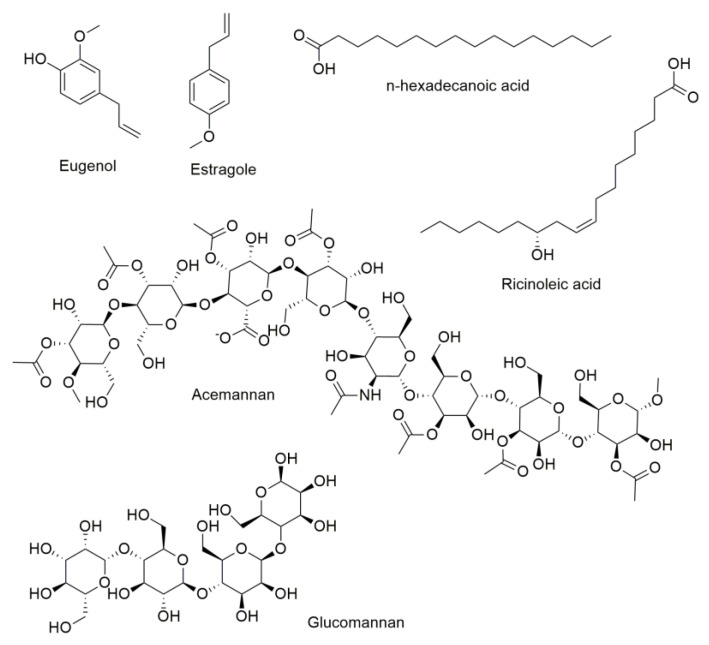
Chemical structures of compounds found in *O. basilicum* (n-hexadecanoic acid, eugenol, and estragole), *R. communis* (ricinoleic acid) and *A. vera* (acemannan and glucomannan).

**Figure 4:**
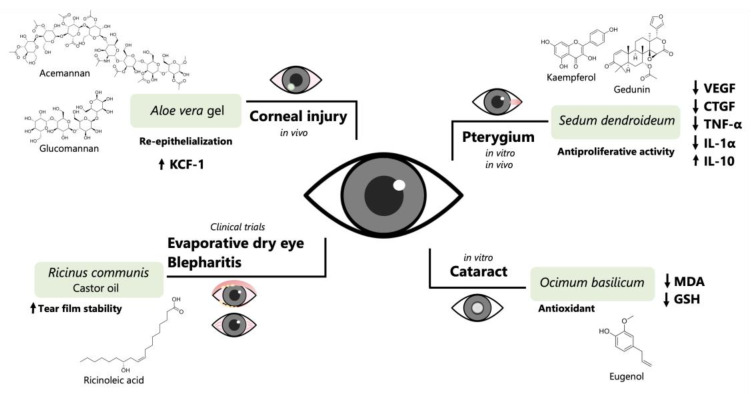
The proposed therapeutic approaches of medicinal plants and their main components in eye conditions. Pharmacological studies point towards antioxidant, antiproliferative, and anti-inflammatory activities as well as corneal wound healing and improvement in tear film stability. VEGF: Vascular endothelial growth factor; CTGF: connective tissue growth factor; TNF-α: tumor necrosis factor (TNF)-alpha; IL-10: interleukin-10; MDA: malondialdehyde; GSH: glutathione (GSH); KGF-1: keratinocyte growth factor-1.

The underlying principle of the naphthalene-induced in vivo cataract model is based on the toxicity in the lens produced by 1,2-naphthalenediol, a reactive metabolite derived from the biotransformation of naphthalene by hepatic cytochrome P450 (CYP) [106], whereas selenite disrupts the antioxidant defense mechanism of the lens by reducing GSH levels and impairs calcium homeostasis, leading to nuclear cataract formation [107]. The plants possessing anticataract activity are *E. hirta*, *O. basilicum*, and *C. aurantium*. The anti-cataract activity for these plants involves decreasing the opacity index and/or preventing peroxidative damage or enhancing the glutathione GSH levels. *O. basilicum* displays several pharmacological properties, among them antioxidant, analgesic, anti-inflammatory, antimicrobial, anti-neoplastic, anticancer, anti-osteoporotic, anti-ulcer, neuroprotective, immunomodulatory, hypoglycemic and hypolipidemic, and different bioactive chemical compounds have been identified from this plant [108]. Anand et al. [101] designated n-hexadecanoic acid, eugenol, and estragole (Figure 3) as major antioxidant components in the methanolic extract of *O. basilicum*. Eugenol has been associated with anti-inflammatory, anti-viral, anti-cancer, and cardioprotective activities (Figure 4). Multiple mechanisms of action in the inflammatory process have been described in eugenol, for example, inhibition of cyclooxygenase activity [108]; reducing myeloperoxidase activity, TNF-α levels, NF-κB expression, and lipid peroxidation malondialdehyde (MDA); and increased GSH levels [109]. The MDA reduction and GSH increase were observed in selenite-induced cataractogenesis in rat lenses in the presence of methanolic extract of *O. basilicum* [101]. Thus, eugenol could be related to this effect, but more detailed experiments should be performed to confirm this hypothesis. 

There are models for assessing the IOP-lowering effect of plants, including the water-loading model and the steroid-induced IOP. The water-loading model (acute IOP elevation) is used to mimic primary angle closure, whereas the administration of corticosteroids such as prednisolone can lead to the development of IOP resembling the primary open angle in humans (chronic IOP elevation) [110]. Due to their IOP-lowering effects, *D. carota* and *O. basilicum* are plant species with anti-glaucoma potential. In traditional Mexican medicine, fresh leaves of *D. carota* have historically been used for addressing “eye problems”, although without specifying eye conditions. Two studies have investigated the effects of the seeds and roots of this plant, demonstrating their potential in reducing intraocular pressure (IOP) and mitigating retinal damage in diabetic rats, respectively, as outlined in Table 2. However, there is a lack of research focusing on the leaves of this plant. Considering the results presented by Agarwal et al. [89] and El-Mansi et al. [90] on seeds and roots, it is imperative to initiate investigations into the potential effects of *D. carota* leaves. This research would serve to validate the traditional use of these leaves for eye health among the Mexican population. A similar situation arises with *C. roseus*, a well-known plant marked for its diverse medicinal benefits; it is rich in alkaloids with anticancer activity. The roots of this plant are employed in traditional medicine to treat conjunctivitis. However, there is limited evidence on the advantages of methanol leaf extract in preventing alkali burn-induced corneal neovascularization in rabbits. Comprehensive examinations, including chemical, pharmacological, and toxicological studies, are warranted for *C. roseus*, with special attention to its alkaloid content [111].

*A. vera* and *R. communis* have been assessed in clinical trials of ocular ailments. *A. vera* is well-known as a bioactive natural product. Preclinical studies on the effect of *A. vera* described in Table 2 have revealed its potential to improve eye health. This is a plant used worldwide for therapeutic, pharmaceutical, and commercial applications. *A. vera* is one of the most ancient and well-documented medicinal plants and has been considered a “healing plant” due to its variety of medicinal properties. *A. vera* is composed mainly of polysaccharides, proteins, minerals, and water [112]. Traditionally, the leaf gel of *A. vera* has been used to treat wounds and other skin problems; its wound-healing properties are attributed to acemannan and glucomannan (Figure 3), two polysaccharides produced in the leaf gel [112,113]. Although *A. vera* gel is used extensively for treating dermal wounds, few studies have assessed this effect on corneal injury, and the molecular mechanisms underlying this beneficial effect are associated with the re-epithelialization process (Figure 4). In alkali-burned corneas in in vivo models of normal rabbits and rats as well as diabetic rats, topical application of *Aloe vera* gel promotes corneal wound healing by enhanced corneal re-epithelialization. In this process, many growth factors secreted from fibroblasts, such as the keratinocyte growth factor-1 (KGF-1), play an important role in re-epithelialization. It has been demonstrated that acemannan stimulated KGF-1 synthesis from gingival fibroblasts [114]. Special attention should be given to acemannan and glucomannan in further studies to identify their roles in treating eye conditions. *R. communis* showed therapeutic potential in managing ocular surface disease in clinical trials. A recent review reported the ocular surface benefits of using castor oil, derived from the bean of *R. communis*, in six different clinical trials [99]. Castor oil has demonstrated effectiveness in meibomian gland dysfunction and improvement of the ocular symptoms and signs of blepharitis. Clinical trials reported greater stability and less evaporation of tears after the ophthalmic use of castor oil for approximately 30 days. Due to the hydrophilic and hydrophobic characteristics of castor oil, the mechanism of action is related to stabilizing the tear film, and ricinoleic acid is proposed to participate in this process (Figure 4). Therefore, castor oil is a potential treatment for evaporative dry eye [99].

Indeed, the potential adverse effects of the medicinal plants used in self-care or accidental contact with the eyes should not be overlooked. Many of these plants show promising pharmacological activities, but they may also have the potential to cause ocular injuries or discomfort [115,116]. For instance, *A. mexicana* and *S. dendroideum* have been reported to induce burning sensations in the eyes when used topically [28,38]. Such adverse reactions highlight the need for cautious and informed use of these plants for ocular conditions. Therefore, it is crucial to assess toxicological studies, particularly with members of the *Euphorbia* genus and other plant species known to contain milky sap or latex, which have been associated with keratouveitis from accidental ocular exposure [117,118]. The combination of pharmacological efficacy and safety assessment will be pivotal in determining which medicinal plants can be effectively and safely used as complementary or alternative therapies for ocular conditions. Collaborative efforts between traditional medicine practitioners, ethnobotanists, toxicologists, and the scientific community will be crucial to ensuring a holistic approach to evaluating the potential of medicinal plants for ocular health, and ultimately improving patient care.

## 3. Future Perspectives

Traditional medicine has a long history of utilizing medicinal plants for various health conditions, including eye ailments. It is crucial to integrate traditional knowledge with scientific evidence and clinical research. This collaborative approach can lead to the development of novel and effective treatments for ocular disorders, ultimately enhancing eye health and patient well-being.

Given the promising findings from ethnobotanical surveys and preclinical studies, it is reasonable to consider advancing research on *Sedum* species for potential preclinical studies and toxicological assessments. These investigations can provide valuable insights into the safety and efficacy of *Sedum*-based treatments for ocular diseases, further supporting their potential as alternative or complementary therapies for eye health. Indeed, future pharmacological studies focusing on eye ailments should pay particular attention to plants from the Crassulaceae and Euphorbiaceae families, including members of the *Sedum* genus, which are commonly known as succulents. These plants have shown promising pharmacological activity and have been traditionally used to treat eye conditions. Investigating their potential therapeutic effects and identifying active compounds could lead to the development of novel and effective treatments for various eye diseases. The isolation and elucidation of the structure of the bioactive compounds used for improving eye health remain to be performed. In addition, it is necessary to elucidate the molecular mechanisms and perform toxicological studies with plant extracts and their active compounds which are used for treating eye conditions. These studies are scarce and are needed to assess their pharmacological effects in clinical trials.

As traditional medicine continues to be an important part of healthcare in several communities in Mexico, it is equally crucial to conduct thorough toxicological studies using in silico and in vitro (i.e., HaCaT cells, two- and three-dimensional cell culture models, and other cell lines) approaches. The use of in vitro and in silico studies can help to reduce the reliance on animal testing, providing more accurate and relevant results. These studies are essential to ensuring the safety of applying medicinal plants to the eye and informing people about the potential risks or adverse effects. Taking into consideration the potential hazards and verifying the safety of traditional remedies through modern scientific methods is paramount to protecting the ocular health of those relying on these practices. In addition, stability tests should be performed on pharmaceutical formulations containing plant extracts and plant-derived compounds. These tests are necessary because half of the pharmacological studies were conducted with drops containing plant extracts. The chemical standardization of these pharmaceutical preparations is crucial for the herbal medicine industry’s ability to provide high-quality products. These preparations should also follow good manufacturing practices throughout their processing. This pharmacological, pharmaceutical, and toxicological information could provide more evidence regarding the potential of medicinal plants from Mexico to be used for treating eye conditions. The integration of medicinal plants into a medical system is highly desirable, especially in low-income areas and rural zones of many countries. The current government in Mexico is promoting the use of medicinal herbs in rural communities. Traditional practitioners are sharing their ancient knowledge of herbal medicine with scientific researchers. The integration of traditional knowledge and modern research methodologies can lead to a better understanding of the benefits and risks associated with using medicinal plants for eye ailments. Collaborative efforts between traditional healers, scientists, and healthcare professionals can facilitate the gathering of valuable data on the safety and efficacy of these remedies. By combining the strengths of traditional medicine with evidence-based research, we can promote the safe and effective use of medicinal plants in eye care. Additionally, this approach can pave the way for the discovery of new therapeutic options and enhance overall eye health in Mexico and other countries.

The evidence of pharmacological effects demonstrated in preclinical studies indicates the promising therapeutic potential of these medicinal plants. As such, further research and exploration of these natural resources could pave the way for the development of new and effective treatments for various eye diseases. It is crucial to recognize the value of traditional medicine and to integrate it into modern healthcare practices, especially in regions where it has been an essential part of the culture and medical heritage.

## 4. Limitations of the Study

In many cases, the plant part used, the method of administration, and more information could not be obtained from the literature which we consulted. The accurate interpretation of old academic books was one of the main challenges of this work. In addition, as mentioned in Section 2.2, only a small number of medicinal plants followed the method of preparation reported by Mexican traditional medicine for treating eye conditions. This indicates the need to perform ethnobotanical studies in Mexico regarding the medicinal flora used for treating eye conditions. More detailed and updated information on the diseases treated with each medicinal plant is needed to plan pharmacological studies.

## 5. Materials and Methods

The data for this study were obtained through an extensive bibliographic search of the published studies related to Mexican ethnobotanical information. The search was conducted using electronic databases, including PubMed, ScienceDirect, and Google Scholar, up until 31 July 2023, without a specific period or language restriction. The search included the following keywords: “medicinal plant”, “ethnomedicinal”, “ocular”, “eye”, “eye diseases”, “eye conditions”, “ophthalmologic problems”, “pterygium”, “cataract”, “conjunctivitis”. Ethnobotanical knowledge was collected from scientific articles, Mexican herbal medicine books, or academic theses, and additional information was identified from the references in the retrieved articles. All ethnobotanical reports were included, even if they did not provide complete data on the preparation, route of administration, plant part used, or specific eye condition. The literature review gathered preclinical and clinical data from medicinal plants with ethnomedicinal purposes for eye health. Only studies focusing on the single use of a medicinal plant were considered. The accepted botanical names of each plant species were validated and updated, if necessary, according to The World Flora Online (http://www.worldfloraonline.org, accessed on 25 September 2023) and Plants of the World Online (https://powo.science.kew.org/, accessed on 25 September 2023). The chemical structures were drawn using the ChemDraw Ultra 12.0 software.

## 6. Conclusions

The rich biodiversity in Latin America, particularly in countries like Mexico, has fostered a strong tradition of using traditional medicine as the primary approach for treating non-severe and acute diseases. Over the centuries, ancient botanical knowledge has provided a wealth of information on the medicinal properties of various plants. This work indicates that there are a limited (11%) number of medicinal plants used for eye conditions with pharmacological studies. This indicates the importance of performing pharmacological and toxicological studies with these plant species.

Eye conditions are often associated with some of the most prevalent diseases in Mexico, such as Type 2 diabetes mellitus. Despite the significance of these eye conditions, the available literature suggests that people’s knowledge about specific eye diseases is limited. Instead, they tend to refer to generic eye problem names when discussing the use of medicinal plants for eye care. Most of the ethnobotanical studies in Mexico on medicinal plants used for eye conditions were documented in the central and southern regions. This review highlights the need to document the traditional knowledge of herbs used for treating eye diseases, especially in the states of Northern Mexico, where this information is scarce. This documentation is essential, as it serves as a reservoir of potential novel treatments for eye diseases.

Due to ethnobotanical information and preclinical findings, members of the *Sedum* genus are a promising alternative for the obtention of new bioactive compounds for treating eye conditions. Phytochemical studies with medicinal herbs are required to obtain new bioactive compounds.

Collaborative efforts between traditional healers, scientists, and healthcare professionals can bridge the gap between traditional knowledge and modern evidence-based medicine, ultimately benefiting the population’s eye health. By combining the wisdom of ancient botanical knowledge with scientific research, we can unlock the full potential of medicinal plants and contribute to the development of more comprehensive and effective eye care treatments in Mexico. 

## Figures and Tables

**Table 1:** Ethnobotanical information on medicinal plants used for eye conditions in Mexico.

Plant Family	Plant Name	Common Name (Spanish or Autochthonal)	Plant Part Used	Medicinal Use	Ethnobotanical Information/Estate	Reference
Anacardiaceae	*Metopium brownei* (Jacq.) Urb.	Che’ chen (Lacandon); Chechem (spanish)	Sap/crude “Bleed sap from the base of the tree”	Eye irritation	“Wash eyelids with sap”/Chiapas	[16]
	*Schinus molle* L.	Pirú (Spanish)	Not specified	Eye cleaner	Not specified/Estado de México	[17]
	*Spondias purpurea* L.	Ciruela (Spanish)	Not specified	Eye pain	Not specified/Yucatán	[18]
Apiaceae	*Daucus carota* L.	Zanahoria (Spanish)	Fresh leaves	Eye problems	Squeezed/Chiapas	[19]
Apocynaceae	*Catharanthus roseus* (L.) G.Don	Vicaria (Spanish and Chontal)	Root	Conjunctivitis	Not specified/Tabasco	[20]
Araceae	*Philodendron sagittifolium* Liebm.	“chapiz grande”, “malaste grande” (Spanish); “tantai” (Totonaca)	Leaves	To cure strabismus	“Leaves applied to eyelids of sleeping children, to cure strabismus”/Puebla	[21]
Arecaceae	*Acrocomia aculeata* (Jacq.) Lodd. ex Mart.	Coyol redondo, palma (Spanish)	Fresh bark	Eye problems	Decoction/Veracruz	[22]
Asphodelaceae	*Aloe vera* (L.) Burm. f.	Sábila (Spanish)	Leaf gel	Eye cleaner	Crushed/Oaxaca	[23]
Asteraceae	*Arnica montana* L.	Arnica (Spanish)	Aerial parts	Improves eyesight	Decoction and topical administration/Puebla	[24]
	*Chamaemelum nobile* (L.) All.	Manzanilla (Spanish)	Whole plant	Eye infection	Oral infusion of whole plant/Guanajuato	[14]
	*Helianthus annuus* L.	Girasol (Spanish)	Fresh leaves	Eye problems	Infusion/Chiapas	[19]
	*M. chamomilla*	Manzanilla (Spanish)	Not specified	Eye cleaner	Decoction, infusion, washes/Oaxaca	[23]
			Not specified	Eye irritation	Not specified/Mexico city	[25]
			Aerial parts	Eye infection	“Tea, the infected eyes are washed with it (topical)”/Puebla	[26]
			Leaves and inflorescences	Eye infection	“Applied as eye drops”/Nuevo León	[27]
			Leaves, whole plant	Conjunctivitis	Infusion, oral/Guerrero	[28]
			Whole plant	Not specified	The infusion is used as eye drops/Nuevo León	[29]
			Leaves and flowers	Eye problems	Not specified/Zacatecas	[30]
			Not specified	Eye irritation	Not specified/Oaxaca	[31]
			Fresh leaves	Eyes problem	Bath/Veracruz	[22]
			Leaves and flowers	Eye cleaner	“Put two warm drops of the 5apónica directly in the eye”/Coahuila	[13]
	*Taraxacum officinale* F.H. Wigg	Diente de león (Spanish)	Aerial part, root	Conjuntivitis	Decoction, oral/Puebla	[24]
	*Zinnia peruviana* (L.) L.	Mal de ojo (Spanish)	Not specified	Eye irritation	Not specified/Guanajuato	[32]
Commelinaceae	*Commelina erecta* L.	“matalín” (Spanish); “kasmalj” (Totonaca)	Sap	Eye cleaner	“Sap used as eyedrops to clean the eyes”/Puebla	[21]
	*Tradescantia spathacea* Sw.	Maguey morado (Spanish)	Leaves	Eye irritation	Infusion of leaves and applied as eye drops/Tabasco	[33]
	*Tradescantia zebrina* var. *zebrina*	Matlali color morado (Spanish); hierba de 1os ojos (Spanish)	Leaves	Cataract	“The juice from its leaves and twigs is put directly over the cataract of the eye”/Oaxaca, Puebla, and Veracruz	[34]
Costaceae	*Costus pulverulentus* C. Presl	Cañita agría (Spanish)	Stem	Eye irritation	Infusion of stem and applied as eye drops/Chiapas	[35]
Crassulaceae	*Echeveria elegans* var. *simulans* Poelln.	Siempre viva (Spanish)	Sap	Red eyes, irritated eyes	Eye drops/Nuevo León	[36]
	*Kalanchoe pinnata* (Lam.) Pers.	Siempre viva, “hoja fresca” (Spanish); “Tkuya tuwan” (Totonaca)	Leaves	Eye cleaner	“Sap of leaves used to clean the eyes”/Puebla	[21]
	*Sedum allantoides* Rose	Cola de Borrego (Spanish)	Sap	Conjuntivitis	The sap of this plant is used as an antiseptic in mild eye infections, conjunctivitis, and children with thrush (*Candida albicans* infection)/Puebla	[37]
	*Sedum dendroideum* Moc. & Sessé ex DC.	Siempre viva (Spanish)	Leaves/sap	Eye pain	Detached leaves are squeezed directly into the eye. A small amount of sap is directly applied to the eye to avoid a burning sensation/Mexico City	[38]
		Siempre viva (Spanish)	Not specified	Eye infection	Crushed/Oaxaca	[23]
	*Sedum diffusum* S. Watson	Chismes (Spanish)	Sap	Red eyes, irritated eyes	Eye drops/Nuevo León	[36]
	*Sedum morganianum* E.Walther	Cola de Borrego (Spanish)	Fresh leaves	Eye infection	Squeeze/Chiapas	[19]
		Iná meda (*Me 'phaa*)	Leaves	“Carnosidad” ^1^ Eye infection	Topical/Guerrero	[39]
	*Sedum oxypetalum* Kunth	Siempre viva (Spanish)	Leaves, stem	“Carnosidad” ^1^, eye cleaner	Extract drops and poultice (Cataplasm)/Mexico City	[25]
	*Sedum praealtum* A.DC.	Siempre viva “damdo” (Spanish)	Leaves	Eye irritation	Not specified/Hidalgo	[40]
		Siempreviva; flor de siempreviva (Spanish); su sá (Ngiba)	Not specified	Eye irritation	Not specified/Oaxaca	[31]
	*Sedum × rubrotinctum* R.T.Clausen	Dedo de niño (Spanish)	Leaves	Eye infection	“Cut leaves and squeeze out the liquid until it contains and apply a few drops in the ear or eye if it is the case”/Puebla	[41]
Euphorbiaceae	*Croton cortesianus* Kunth	Not specified	Sap	Eye infection	Not specified/southeast San Luis Potosi and northern Veracruz	[42]
	*Croton reflexifolius* Kunth	Not specified	Resin	Eye problems	“The resin of *Croton reflexifolius* is used for treating pimples in the mouth (herpes) and eye problems”/Yucatán	[43]
	*Croton repens* Schltdl.	Sangre de grado de la sabana/Soj kobak/Soj muk (Popoluca)	Sap	Retina complication	“The sap of astringent plants is applied to the eye for cleaning the retina”/Veracruz	[44]
	*Euphorbia hirta* L.	Hierba de la golondrina (Spanish)	Stem	Eye irritation	Not specified/Hidalgo	[40]
			Not specified	Conjuntivitis	Not specified/Estado de México	[17]
	*E. prostrata*	Hierba de la golondrina (Spanish)	Whole plant	Cataracts	“An infusion of the whole plant is used for diarrhea and cataracts”/Nuevo León	[29]
			Leaves	Eye diseases	“Two or three leaves of the plant are squeezed, and the fluid is applied to the eyes”/Not specified	[45]
		Golondrina (Spanish)	Leaves	Watering eyes, “nubes” ^2^	Two or three leaves of the plant are squeezed into the eye/Veracruz	[46]
	*J. dioica*	Sangre de drago, sangregado (Spanish)	Aerial parts and roots	Eye irritation	Infusion, oral/Not specified	[45]
		Sangregado (Spanish)	Not specified	Eye irritation, “nubes” ^2^, blindness	Not specified/Guanajuato	[47]
		Sangre de drago, sangre de grado (Spanish)	Sap	Eye cleaner	“Apply a drop of sap to the eye”/Coahuila	[13]
	*Ricinus communis* L.	Higuerilla (Spanish)	Not specified	Conjunctivitis	Not specified/Estado de México	[17]
Fabaceae	*Senna spectabilis* (DC.) H.S. Irwin & Barneby	Flor de todos 1os santos (Spanish)	Flowers	To wash the eyes	“The concoction of the flowers is used to wash the eyes and avoiding ‘evil eye’ (mal de ojo) occurs”/Oaxaca, Puebla and Veracruz	[34]
	*Dalbergia glabra* (Mill.) Standl.	Not specified	Leaves	Eye infections	“Infusion used to treat eye infections”/Yucatán	[48]
	*Gliricidia sepium* (Jacq.) Kunth	Cocohite (Spanish); aj chánté (Chontal)	Leaves	Conjunctivitis	Not specified/Tabasco	[20]
	*Prosopis laevigata* (Humb. & Bonpl. ex Willd.) M.C.Johnst.	Mezquite (Spanish)	Leaves	Eye problems	“Tea from leaf shoots, the eyes are washed”/Puebla	[26]
		Mezquite (Spanish); Mizquitl (Nahuatl)	Leaves	Eye infection	Infusion, oral/Guerrero	[28]
	*Senna racemosa* (Mill.) H.S.Irwin & Barneby	Not specified	Bark	Eye infections	“Infusion is used to treat eye infections”/Yucatán	[48]
Fouquieriaceae	*Fouquieria diguetii* (Tiegh.) I.M.Johnst.	Palo Adán (Spanish)	Flower’s sap	Cataract	“Applied directly to the eye”/Baja California Sur	[49]
Lamiaceae	*Agastache mexicana* (Kunth) Lint & Epling.	Toronjil (Spanish)	Not specified	Eye cleaner	Infusion, washes/Oaxaca	[23]
	*Lavandula angustifolia* Mill.	Lavanda (Spanish)	Leaves	Conjunctivitis	Infusion, oral/Puebla	[50]
	*Ocimum basilicum* L.	Albahaca (Spanish)	Not specified	Eye pain	Not specified/Yucatán	[18]
		Albahacar (Spanish); Albajaka (Chontal)	Aerial part	Conjunctivitis	Not specified/Tabasco	[20]
	*Ocimum carnosum* (Spreng.) Link & Otto ex Benth	Siempreviva (Spanish)	Leaves	Cataract	Maceration, topical/Hidalgo	[51]
	*Rosmarinus officinalis* L.	Romero (Spanish)	Aerial part	Blurry vision	Decoction, infusion, crushed; oral/topical/Puebla	[24]
	*Salvia hispanica* L.	Chía (Spanish)	Seeds	“Basura en el ojo” (foreign body sensation)	Seeds are mashed and put into the eye. Tear production helps to eliminate the foreign body sensation/Guerrero	[52]
Lauraceae	*Persea americana* Mill.	Aguacate (Spanish); tchunue (Ngiba)	Not specified	Eye irritation	Not specified/Oaxaca	[31]
Malvaceae	*Malvaviscus arboreus* Dill. ex Cav.	Sibí; Sibil (Spanish); Yopo 'aj ts 'ibi (Chontal)	Leaves	“Carnosidad” ^1^	Not specified/Tabasco	[20]
	*Pachira aquatica* Aubl.	Zapote de agua (Spanish); Ajp 'o 'te c (Chontal)	Cortex	Conjuntivitis	Not specified/Tabasco	[20]
Papaveraceae	*Argemone mexicana* L.	Tachina, Táchino (Mayo); Xazácös; (Seri); Cardo (Spanish); Chicalote (Spanish)	Shoots	Eye infection	Decoction/Sonora	[53]
		Chicale (Spanish); Chicalotl (Nahuatl)	Flower, latex	Eye infection	Maceration, topical/Guerrero	[28]
	*Argemone ochroleuca* Sweet	Chicalote (Spanish)	Not specified	Eye infection, cataracts	Infusion, washes/Oaxaca	[23]
			Flowers, latex	“Carnosidad” ^1^, eye irritation	Extract drops/Mexico City	[25]
		Chicatl (náhuatl)	Latex	“Carnosidad” ^1^	“1 or 2 drops of latex into the eye. To improve effectiveness, 1 drop is recommended before going to sleep/Guerrero	[52]
	*Argemone platyceras* Link & Otto	Chicalote blanco (Spanish)	Flowers, latex	“Carnosidad” ^1^,eye irritation	Extract drops/Mexico city	[25]
		Chicalote (Spanish)	Not specified	Eye pain	Not specified/Estado de México	[17]
Plantaginaceae	*Plantago major* L.	Lantén (Spanish)	Not specified	Eye cleaner	Washes/Oaxaca	[23]
Polypodiaceae	*Pleopeltis polypodioides* (L.) E.G. Andrews & Windham	Siempre viva (Spanish)	Aerial part	Conjuntivitis	Infusion, oral/Puebla	[24]
Rhamnaceae	*Sarcomphalus obtusifolius* (Hook. ex Torr. & A.Gray) Hauenschild	Jutuqui, Jo’otoro (Mayo); Bachata, Ciruela del monte, Hui-chillame (Spanish)	Shoots	Eye infections	Decoction/Sonora	[53]
Rosaceae	*Rosa* sp.	Rosa de castilla (Spanish)	Flowers	Eye cleaner	“Boiling two to three roses and wash the eyes with the infusion”/Veracruz	[46]
	*Rosa × centifolia*	Not specified	Petal; flower	Ophthalmological problems	Not specified/Oaxaca	[54]
		Rosa de Castilla (Spanish)	Flowers		“Tea (oral) and used to wash the eye (topical)”/Puebla	[26]
			Not specified	Eye irritation	Not specified/Oaxaca	[31]
			Not specified	Eye irritation	Not specified/Mexico city	[25]
	*Rosa chinensis* Jacq.	Rosa concha (Spanish)	Flowers	To reduce swelling	“The concoction of the flower is put directly over the eyes to reduce swelling”/Oaxaca, Puebla, and Veracruz	[34]
		Rosa (Spanish)	Not specified	Eye problems	Not specified/Estado de México	[17]
	*Rosa gallica* L.	Rosa de castilla (Spanish)	Not specified	Conjunctivitis; improves eyesight; eyes wash	Infusion, washes/Oaxaca	[23]
		Rosa de castilla (Spanish); Nich i castilla (Chontal)	Flowers	“Carnosidad” ^1^	Not specified/Tabasco	[20]
		Rosa de castilla (Spanish)	Flowers	Eye infection	Infusion, washes/Guanajuato	[14]
	*Rosa moschata* Herrm.	Flor de concha, Rosa Concha (Spanish); U nich pat (Chontal)	Flowers	“Carnosidad” ^1^,eye irritation	Not specified/Tabasco	[20]
	*Rosa multiflora* Thunb.	Rosa blanca (Spanish)	Flowers	Eye irritation	Infusion, vaporization, topical/Puebla	[24]
Rutaceae	*Citrus × aurantiifolia* (Christm.) Swingle	Limón (Spanish)	Leaves; fruit	Conjunctivitis	Infusion, essential oil/oral/Central and southern Mexico	[45]
	*Citrus × aurantium* L.	Limonero (Spanish)	Not specified	Eye cleaner	Infusion, washes/Oaxaca	[23]
	*Citrus × limon* (L.) Osbeck	Lima chichi (Spanish)	Fresh fruit	Infection	“Infection in the eye” Squeezed/Veracruz	[22]
	*Ruta chalepensis* L.	Ruda (Spanish)	Whole plant	“Vista venteada”	“The powdered plant is mixed with aguardiente, an alcoholic beverage, and camphor, the resultant paste is applied close to the eyes to treat blurred sight”/Querétaro	[55]
Solanaceae	*Capsicum baccatum* L.	Hoja de chile (Spanish)	Aerial parts; fruit; leaf	Ophthalmological problems	Not specified/Oaxaca	[54]
Viburnaceae	*Sambucus mexicana* C.Presl ex DC.	Not specified	Flower; leaf	Ophthalmological problems	Not specified/Oaxaca	[54]
Vitaceae	*Cissus verticillata* subsp. *verticillata*	Not specified	Aerial parts; fruit	Ophthalmological problems	Not specified/Oaxaca	[54]
	*Vitis tiliifolia* Humb. & Bonpl. ex Schult.	Bejuco de uva (Spanish)	Leaves	Eye cleaner	“Boiling the leaves with water, the infusion is used to wash the eyes”/Querétaro	[55]
		Uva silvestre (Spanish)	Not specified	“Carnosidad” ^1^	Not specified/Estado de México	[17]
Zingiberaceae	*Hellenia speciosa* (J. Koenig) S.R. Dutta	Päsak (Lacandon); Jengibre (Spanish)	Sap/crude “Cut stalk and drain sap”	Eye irritation	“The sap is directly applied to eyes”/Chiapas	[16]

^1^ Growth of conjunctival tissue over the cornea; ^2^ corneal opacities.

**Table 2:** Pharmacological studies (in vitro, in vivo, and clinical trials) of medicinal plants used for eye conditions in Mexico.

Species Name	Extract	Vehicle or Formulation	Study/Model or Clinical Intervention	Way of Administration	Outcome	Reference
*D. carota*	Aqueous seed extract	Dried and powdered extract was dissolved in 0.25% hydroxy propyl methylcellulose	In vivo/water-loading model and steroid induced model IOP in rabbits	Topically (instilled) 50 µL	IOP reduction of 29.39% (water-loading model) and 30.27% (steroid-induced model) at 0.6%.	[89]
	Chloroform root extract	-	In vivo/STZ-induced diabetic retinal damage in rats	Orally administered at 200 mg/kg/day	↑ Serum plasma retinol (~1.70%). Protection and attenuation of retinal damage via downregulation of apoptotic pathways, enhancing oxidative capacity, and modulation of retinal neurotransmission at 200 mg/kg/day.	[90]
*C. roseus*	70% methanol leaf extract	Eye drops (1% *w*/*v*) were prepared in normal saline and DMSO (4%)	In vivo/alkali burn-induced corneal neovascularization in rabbits	Topically applied (instilled) three eye drops three times a day	↓ Vessel length and thickness; their propagation towards the cornea was stopped.	[91]
*A. vera*	Leaf gel	*Aloe vera* gel-derived eye drops	In vivo/alkali-burned corneas in rabbits	Topically (instilled) eye drops four times a day for seven days	↓ Corneal epithelial defect area; ↓ rate of keratocyte loss.	[92]
		Eye drops, Gel lyophilized powder (60 mg/mL) in saline.	In vivo/alkali-burned corneas in normal and diabetic rats	Topically applied (instilled) eye drops four times daily for 3 days	Promoting corneal wound healing through facilitating re-epithelization and reducing inflammation in diabetic rats.	[93]
		Eye drops, gel	In vivo/mechanically induced corneal epithelial lesion in rabbits.	Topically applied (instilled) 50 µL eye drops applied three times daily	No effect	[94]
		6 and 12% gel solutions with potassium sorbate as a preservative	In vivo/methylcellulose-induced ocular hypertension in rabbits	Topically applied (instilled) eye drops applied every 8 h for 2 days	IOP reduction of 8.6 and 10.4% with 6 and 12% gel solutions, respectively	[95]
	Ethanol, ethyl acetate, and heptane extracts	Dissolved in DMSO	In vitro/human normal corneal cell line 10.014 pRSV-T (ATCC No. CRL-11515)	-	↓ NO production ↓ IL-1β, IL-6, TNF-α and IL-10 production	[96]
	Methanol extract from fresh leaves	Lyophilized powder (100 μg/mL)	In vitro/epithelial adenovirus 12-SV40 hybrid-transformed HCE cells (ATCC^®^ CRL-11135™); exposure to H_2_O_2_	-	↑ Cell viability ↓ ROS production and MDA levels ↑ Gene expression of Nrf2, SOD2 and Catalase ↓ COX-2, TNF-α, IL-6 and IL-1β mRNA expression	[97]
*S. dendroideum*	Dichloromethane extract from leaf juice; lyophilized powder	20 mg/mL dichloromethane extract dissolved in vehicle (cyclodextrin and Tween 20 solution)	In vivo/tetradecanoylphorbol acetate-induced pterygium-like eye lesion in mice	Topically applied (instilled) daily for fifteen days	↓ Corneal opacity ↓ TNF-α and IL1β ↑ IL-10	[85]
	The stems and leaves were blended with distilled H_2_O	100 mg of lyophilized powder in 1 mL of DMSO	In vitro/human pterygium fibroblast primary culture	-	↓ Proliferation ↓ VEGF and CTGF expression at 250 μg/mL	[86]
*E. hirta*	Ethanolic extract of aerial parts	-	In vivo/naphthalene-induced cataract in rats	Orally for 28 days	↓ Opacity index at 200 and 400 mg/kg	[98]
*R. communis*	Castor oil	Castor oil emulsions	Six clinical trials ^1^/meibomian gland dysfunction; dry eye; contact lens discomfort; blepharitis	Topically applied (instilled) eye drops	Beneficial effects on the lipid layer, tear film integrity, eyelash health, and meibomian gland functionality	[99]
*O. basilicum*	Aqueous extract from seeds	Extract in 0.25% Hydroxypropyl methylcellulose	In vivo/water-loading and steroid-induced ocular hypertension in rabbit eyes	Topically applied (instilled) eye drops, single drop of extract	IOP reduction at 0.5% of extract	[100]
	Methanolic leaves extract	200 μg/mL DMEM	In vitro/selenite-induced cataractogenesis in rat lenses	-	Prevented selenite-induced cataract formation. ↑ GSH level ↓ MDA	[101]
	Methanolic leaves Extract	200 μg/mL DMEM	In vitro/selenite-induced cataractogenesis in rat lenses	-	Prevented alterations of the insoluble-to-soluble-protein ratio and the protein carbonyl and sulfhydryl levels. Prevented the reduction in mRNA transcript levels of the αA- crystallin and βB1-crystallin genes	[102]
*C. aurantium*	Hydromethanol peel extract	-	In vivo/naphthalene-induced cataractogenesis in rats	Orally for 28 days	↓ Opacity index at 200 and 400 mg/kg ↓ Carbonyl and ↑ sulfhydryl levels ↓ MDA and LH Restored enzymatic and non-enzymatic antioxidant enzymes	[103]

^1^ For detailed information, consult the review by Sandford et al. [92]. Reduction (↓); increment (↑); intraocular pressure (IOP); nitric oxide (NO); interleukin-1beta (IL-1β); interleukin- 6 (IL-6); tumor necrosis factor (TNF)-alpha (TNF-α); interleukin-10 (IL-10); reactive oxygen species (ROS), malondialdehyde (MDA); nuclear factor E2-related factor 2 (Nrf2); superoxide dismutase-2 (SOD2); cyclooxygenase-2 (COX-2); vascular endothelial growth factor (VEGF); connective tissue growth factor (CTGF); glutathione (GSH); lipid hydroperoxides (LH).

## Data Availability

Not applicable.
